# US Cystic Fibrosis Foundation and European Cystic Fibrosis Society consensus recommendations for the management of non-tuberculous mycobacteria in individuals with cystic fibrosis: executive summary

**DOI:** 10.1136/thoraxjnl-2015-207983

**Published:** 2016-01

**Authors:** R Andres Floto, Kenneth N Olivier, Lisa Saiman, Charles L Daley, Jean-Louis Herrmann, Jerry A Nick, Peadar G Noone, Diana Bilton, Paul Corris, Ronald L Gibson, Sarah E Hempstead, Karsten Koetz, Kathryn A Sabadosa, Isabelle Sermet-Gaudelus, Alan R Smyth, Jakko van Ingen, Richard J Wallace, Kevin L Winthrop, Bruce C Marshall, Charles S Haworth

**Affiliations:** 1 Cambridge Institute for Medical Research, University of Cambridge, Cambridge, UK; 2 Cambridge Centre for Lung Infection, Papworth Hospital, Cambridge, UK; 3 Cardiovascular and Pulmonary Branch, National Heart, Lung, and Blood Institute, NIH, Bethesda, Maryland, USA; 4 Department of Pediatrics, Columbia University Medical Center, Pediatric Infectious Diseases, New York, New York, USA; 5 Division of Mycobacterial and Respiratory Infections, National Jewish Health, Denver, Colorado, USA; 6 INSERM U1173, UFR Simone Veil, Versailles-Saint-Quentin University, Saint-Quentin en Yvelines, France; 7 AP-HP, Service de Microbiologie, Hôpital Raymond Poincaré, Garches, France; 8 Department of Medicine, National Jewish Health, Denver, Colorado, USA; 9 Division of Pulmonary and Critical Care Medicine, The University of North Carolina at Chapel Hill, Chapel Hill, North Carolina, USA; 10 Department of Respiratory Medicine, Royal Brompton Hospital, London, UK; 11 Department of Respiratory Medicine, Freeman Hospital High Heaton, Newcastle, UK; 12 Department of Pediatrics, University of Washington School of Medicine, Seattle, Washington, USA; 13 The Dartmouth Institute for Health Policy and Clinical Practice, Geisel School of Medicine at Dartmouth, Lebanon, New Hampshire, USA; 14 Department of Pediatrics, Sahlgrenska University Hospital, Gothenburg, Sweden; 15 Service de Pneumo-Pédiatrie, Université René Descartes, Hôpital Necker-Enfants Malades, Paris, France; 16 Division of Child Health, Obstetrics & Gynaecology, University of Nottingham, Nottingham, UK; 17 Department of Medical Microbiology, Radboud University Medical Center, Nijmegen, The Netherlands; 18 Department. of Microbiology, University of Texas Health Science Center, Tyler, Texas, USA; 19 Divisions of Infectious Diseases, Public Health and Preventive Medicine, Oregon Health and Science University, Portland, Oregon, USA; 20 Cystic Fibrosis Foundation, Bethesda, Maryland, USA

**Keywords:** Atypical Mycobacterial Infection, Cystic Fibrosis, Respiratory Infection

## Abstract

Non-tuberculous mycobacteria (NTM) are ubiquitous environmental organisms that can cause chronic pulmonary infection, particularly in individuals with pre-existing inflammatory lung disease, such as cystic fibrosis (CF). Pulmonary disease (PD) caused by NTM has emerged as a major threat to the health of individuals with CF, but remains difficult to diagnose and problematic to treat. In response to this challenge, the US Cystic Fibrosis Foundation (CFF) and the European Cystic Fibrosis Society (ECFS) convened a panel of 19 experts to develop consensus recommendations for the screening, investigation, diagnosis and management of NTM-PD in individuals with CF. PICO (population, intervention, comparison, outcome) methodology and systematic literature reviews were employed to inform draft recommendations, which were then modified to achieve consensus and subsequently circulated for public consultation within the USA and European CF communities. We have thus generated a series of pragmatic, evidence-based recommendations as an initial step in optimising management for this challenging condition.

## Background

Non-tuberculous mycobacteria (NTM) are increasingly being isolated from the sputum of adults and children with cystic fibrosis (CF) both in North America and Europe. Estimates of the prevalence of NTM in the CF population have ranged from 1.3% in the earliest study reported in 1984^1^ to 32.7% in a review of patients with CF over age 40 in Colorado.^2^

The NTM species most commonly identified in individuals with CF from North America and Europe are the slow growing *Mycobacterium avium* complex (MAC, including *M. avium*, *M. intracellulare* and *M. chimaera*), which can be found in up to 72% of NTM-positive sputum cultures, and the rapid growing *M. abscessus* complex (comprising the subspecies *M. abscessus subsp abscessus* (*M. a. abscessus)*, *M. a. bolletii* and *M. a. massiliense* (the latter currently classified as part of *M. a. bolletii*)), which in many centres has now become the most common NTM isolated from individuals with CF.

There has been a rise in the prevalence of NTM-positive cultures in respiratory samples from individuals with CF over the last three decades, which probably reflects a true increase in the frequency of NTM infection. A number of CF studies (eg, Renna *et al*^3^) show year-on-year increases in NTM-positive cultures with no change in surveillance intensity or culture methodology.

Possible reasons for increased NTM-positive cultures in individuals with CF include: increases in environmental exposure to NTM through more permissive temperature settings of home water heaters and more contact with shower aerosols, increased antibiotic usage creating more NTM permissive lung niches, greater chronic use of medications that might impair host immunity to NTM^3^ and/or spread of NTM through person-to-person transmission.^4^

NTM can cause progressive inflammatory lung damage, a condition termed ‘NTM pulmonary disease’ (NTM-PD), which is defined by the presence of specific microbiological, clinical and radiological features.^5^ However, it has become clear that NTM can also transiently, intermittently or permanently reside within the lungs of CF individuals without causing NTM-PD, thus representing asymptomatic infection and creating considerable difficulties in deciding how best to screen for and diagnose NTM.

Further challenges exist in knowing how best to identify NTM in respiratory samples, when and how to initiate treatment for NTM-PD and how NTM may impact individuals under consideration for lung transplantation. As a consequence, the Cystic Fibrosis Foundation (CFF) and European Cystic Fibrosis Society (ECFS) sought to generate a consensus recommendations document to support and standardise the management of NTM infection in children and adults with CF, permitting prospective evaluation of current best practice and forming a foundation for future research programmes.

## Methods

The CFF and the ECFS invited experts to participate in the statement development process. The 19 member committee consisted of professionals with expertise in CF and NTM and included adult and paediatric CF physicians, lung transplant physicians, microbiologists, infectious disease specialists and a parent of an individual with CF. The committee convened in May 2012 and divided into five subgroups, each responsible for a specific topic: epidemiology and risk factors, screening, microbiology, treatment and transplantation. Each subgroup developed topic-specific questions using the PICO format (population, intervention, comparison, outcome). Questions were reviewed and approved by the entire committee before systematic literature searches were conducted.

The members of each subgroup used the PICO questions to guide literature searches in PubMed. Searches were limited to English language and the period 1984–2013. Subgroup members also searched for topic relevant guidelines through searches of the ATS website, the IDSA website, the Clinical Laboratory Standards Institute website and the UK CF Trust website.

After reviewing relevant literature and existing guidelines, subgroup members drafted recommendation statements. In October 2012, a second meeting was convened, and subgroups finalised draft recommendation statements. The committee also voted to set 80% agreement of all 19 members as the threshold for acceptance of a recommendation statement.

Each subgroup submitted final draft questions for entry into an electronic survey tool (Survey Monkey) for the purposes of anonymous voting and comment by all members. A project coordinator administered the survey, and committee members were asked to rate each statement on a scale of 0 (completely disagree) to 9 (completely agree), with 80% or between 7 and 9 being considered ‘good’ agreement. Space for entering free text was also provided after each statement to allow members to cite literature in support of their opinions or suggested revisions. All committee members were required to vote on each statement regardless of their role or expertise. Multiple rounds of voting and revisions to the statements were conducted, and for each round committee members were requested to complete their voting within 3 weeks. The committee chairs reviewed the results from each round and updated the statements based on comments entered by respondents for subsequent rounds.

A draft of the recommendations was presented at the 2013 North American Cystic Fibrosis Conference and the ECFS Meeting. In addition, the committee solicited feedback from the CF communities in the USA and Europe, which included physicians, nurses, physical and respiratory therapists, parents and individuals with CF. Comments collected from this process were considered by the committee in the development of the final recommendation statements.

## Results

**Figure 1:**
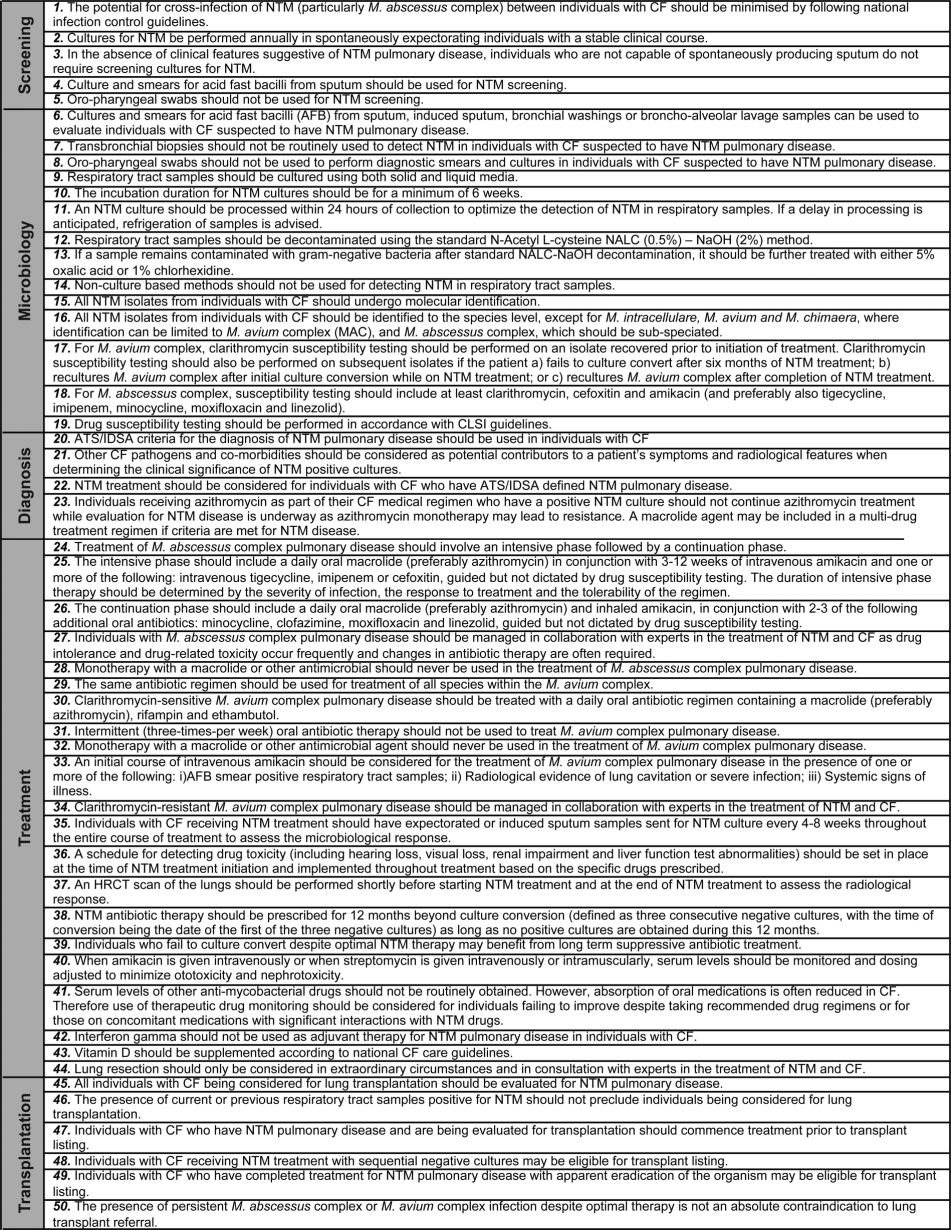
Cystic Fibrosis Foundation and European Cystic Fibrosis Society recommendations on non-tuberculous mycobacteria (NTM) management in cystic fibrosis (CF).

Three rounds of voting were conducted to achieve 80% consensus for each statement. Fifty-three statements were included in the first round of voting and 50 statements in the second and third rounds. Final statements are shown in figure 1.

## Discussion

The management of individuals with CF infected with NTM is extremely challenging. The limited amounts of published research and clinical trial data provide inadequate evidence to base management decisions on how best to screen, diagnose, detect and treat NTM-PD. As a response to this urgent clinical need, the CF Foundation and ECFS formed a committee of clinicians, scientists and infectious disease experts to develop recommendations to guide and assist clinicians in the management of NTM-PD in individuals with CF. The committee believe these recommendations should serve as a benchmark for current medical care while providing a framework to inform the development of clinical, translation and basic research studies to generate robust evidence to base future iterations of these management guidelines leading to better outcomes for individuals with CF infected with NTM.
